# Complex Regional Pain Syndrome: a cross-sectional study of physical symptoms, disability, and psychological health in long term

**DOI:** 10.1097/PR9.0000000000001180

**Published:** 2024-09-20

**Authors:** Ellen Lyckegård Finn, Astrid Parinder, Erika Nyman, Lars. B. Dahlin

**Affiliations:** a Department of Translational Medicine—Hand Surgery, Lund University, Malmö, Sweden; b Department of Hand Surgery, Skåne University Hospital, Malmö, Sweden; c Department of Biomedical and Clinical Sciences, Linköping University, Linköping, Sweden; d Department of Hand Surgery, Plastic Surgery and Burns, Linköping University Hospital, Linköping, Sweden

**Keywords:** Neuropathic pain, Complex regional pain syndrome, Pain catastrophizing, Anxiety, Depression, Psychological health

## Abstract

**Introduction:**

Knowledge about long-time residual symptoms, disabilities, and psychological health in complex regional pain syndrome (CRPS) is limited.

**Objectives:**

The aim was to evaluate outcome, focusing on physical symptoms, disability, and psychological health, in individuals with CRPS through a cross-sectional survey study.

**Methods:**

Individuals with a confirmed diagnosis of CRPS were identified through medical charts and sent validated survey forms (Disabilities of the Arm, Shoulder and Hand—Quick version, Specific Hand Surgery Questionnaire-8 questions, EuroQol 5 Dimensions 3 levels, Life Satisfaction Questionnaire-11, Hospital Anxiety and Depression Scale, Pain Catastrophizing Scale, and Sense of Coherence-29) and complementary questions.

**Results:**

Responders (response rate: 99/238, 42%; CRPS type 1: 72%; CRPS type 2: 28%; time since diagnosis median: 59 [34–94] months) reported remaining symptoms and disability (Disabilities of the Arm, Shoulder and Hand—Quick version score: 45 [20–70]) and more improvement in type 1 than in type 2. Only 9% of individuals with CRPS reported no residual pain or discomfort. Approximately 60% had problems in daily activities, 49% had sleeping problems, and 90% experienced moderate-extreme pain with 23% still on sick leave. The Hospital Anxiety and Depression Scale survey revealed significantly higher scores than a Swedish reference population. Individuals with a low Sense of Coherence and high pain catastrophizing had worse disability and were less satisfied with their lives and physical and psychological health. A lower level of education and more anxiety were associated with worsened disability over time.

**Conclusion:**

Individuals with CRPS suffer in the long term from pain, sleeping problems, and limitations in daily activities with occurrence of anxiety and depression, resulting in dissatisfaction with many aspects of their lives. A low Sense of Coherence and high pain catastrophizing are associated with a worse outcome. Biopsychosocial aspects should be addressed in clinical practice.

## Abstract 2

Supplemental Digital Content is Available in the Text.

Physical symptoms, disability, and problems with psychological health persist in long term after complex regional pain syndrome. Sense of coherence and pain catastrophizing influence outcome.

## 1. Introduction

Complex regional pain syndrome (CRPS) is a chronic primary pain disorder with the defined term nociplastic pain (ie, pain duration >3 months; regional rather than discrete distribution; pain not entirely explained by nociceptive or neuropathic pain mechanisms),^25^ which is now reflected in the International Classification of Diseases 11th Revision (ICD-11).^59^ Complex regional pain syndrome has a multifactorial aetiology and complex pathophysiology^24^ with some sex and age characteristics.^14,28,35,49^ Few long-term follow-up studies exist on health and quality of life in concern to management of CRPS,^52,54^ which should apply a biopsychosocial treatment model.^24^ Patients experience pain with difficulties in working, in home life, and with mental health, whereas sometimes medicating with opioids and other neuropathic drugs.^7,22,24,50,58^ Investigating how CRPS affects the patient's mental health and quality of life is relevant addressing those aspects to achieve optimal recovery^3^ comparing type 1 and type 2.^23^

Living with chronic pain can affect a patient's mental health and quality of life associated with depression and anxiety^46^; knowledge of relevance for health care staff. Level of depression or anxiety observed in patients with CRPS is similar to what is observed in patients with low back pain, but disability and pain severity are more strongly associated with psychological factors in CRPS.^6^ The concept that patients with CRPS have “psychogenic pain” or if “psychosocial factors” may predispose for CRPS is not evidence based.^10,11,14^ Fracture severity and presence of rheumatoid arthritis or musculoskeletal comorbidities are common in CRPS type 1, where fracture severity and presence of prominent pain shortly after trauma predicted CRPS development.^9^ Anxiety and depression may be linked to CRPS and associated with poor outcomes but is not a causing factors.^7,11,42,45,58^

Patients with chronic pain from injury are unsatisfied with one's “life as a whole,” which is linked to higher rates of disability, anxiety, depression, and pain.^55^ To our knowledge, CRPS has not been highlighted concerning life satisfaction or sense of coherence (SoC). A low SoC is associated with a higher disability in other conditions,^19,32^ whereas a high SoC is linked to less anxiety and higher function.^41^ Pain catastrophizing is associated with poor outcome of CRPS^23^ and with dysfunction of prefrontal white matter.^36^ Perception of illness, based on Illness Perceptions Questionnaire–Revised, is related to greater pain, disability, and kinesiophobia in CRPS,^3^ but few studies have used SoC-29 to evaluate SoC in CRPS type 1 and type 2. Differentiating CRPS type 1 and type 2, and using questionnaires that cover different aspects, may be a screening tool in planning treatment strategies in relation to biopsychosocial factors as well a further research area.^17,24^

We aimed to conduct a cross-sectional survey to follow-up patients being diagnosed with CRPS type 1 or CRPS type 2 in upper limb, as well as to compare and evaluate outcome and associated factors, including physical symptoms, disability, and psychological health, to understand how patients perceive living with CRPS in long term.

## 2. Materials and methods

### 2.1. Study design and study population

**Figure 1.**
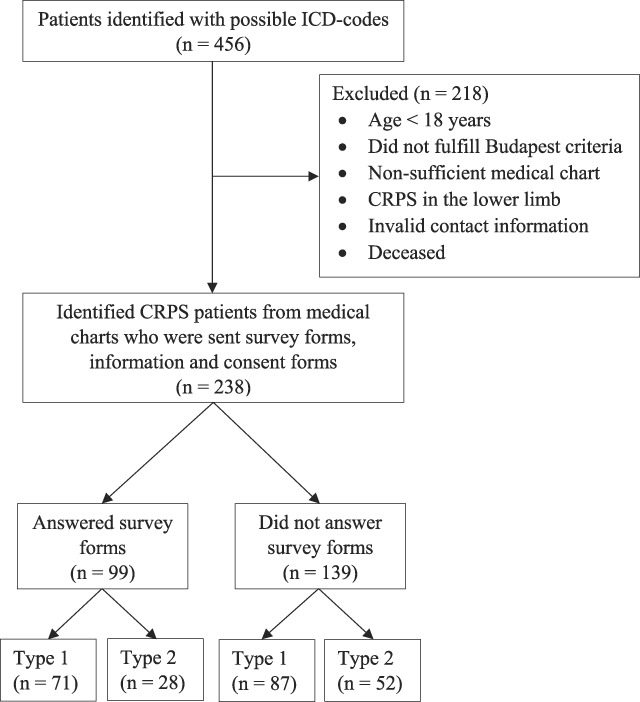
Flowchart of the study population. Flowchart showing a selection of study population from Region Skåne, Sweden, and the Department of Hand Surgery, Plastic Surgery and Burns in Linköping, Sweden, and a number of responders divided in CRPS type 1 and CRPS type 2. CRPS, complex regional pain syndrome.

Individuals treated 2013 to 2020 in the health care sector of Region Skåne, Sweden, and at the Department of Hand Surgery, Plastic Surgery and Burns at Linköping University Hospital, Linköping, Sweden (ICD codes G907, M890, G905, G906, and G564^59^), were evaluated for possible inclusion in the study. Individuals’ medical charts were scanned to evaluate if the individual's medical history fit the Budapest criteria for CRPS with inclusion and exclusion criteria (Fig. 1).^33^ In total, 456 individuals were identified, and 218 individuals were excluded for different reasons (Fig. 1), resulting in 238 eligible and included individuals (Fig. 1). When an individual was identified, their medical chart was examined for sex, age at diagnosis and at follow-up, time since diagnosis, time since end of follow-up, if deceased, and if they had valid contact information. Individuals who were evaluated not to have an apparent nerve lesion, as described, were defined to have CRPS type 1 (n = 158). The individuals were defined as CRPS type 2 if a history of an apparent nerve lesion, nowadays defined as symptoms extending beyond the injured nerve area,^30^ was apparent through the trigger causing CRPS (ie, amputation of part of finger, carpal tunnel syndrome release surgery, other nerve compression lesion, or description of nerve damage in report from acute surgery) or if the individuals’ symptoms clearly suggested damage to a specific nerve (n = 80).

After the examination of medical charts, the 238 included individuals, 83 men (35%) and 155 women (65%), were each sent 7 patient-reported outcome surveys through mail along with information about the study and extra complementary questions (Supplementary material 1, http://links.lww.com/PR9/A239). A consent form was sent out along with the information (received from all individuals).

The questionnaires were Disabilities of the Arm, Shoulder and Hand—Quick version (QuickDASH), Specific Hand Surgery Questionnaire-8 questions (HQ-8), EuroQol 5 Dimensions 3 levels (EQ-5D-3L), Life Satisfaction Questionnaire-11 (LiSat-11), Hospital Anxiety and Depression Scale (HADS), pain catastrophizing scale (PCS), and Antonovsky SoC-29 (SoC-29), which are validated surveys and culturally adapted into Swedish (Supplemental Table 1, http://links.lww.com/PR9/A239).^15,16,38,44,55^ The selected surveys were chosen to evaluate specific symptoms related to the limb/hand, limitations in activities and to get a good overview of the individual's overall health, including mental health, pain catastrophizing, and SoC. The complementary questions consisted of 5 multiple choice questions concerning current disability compared with when it was at its worst (rating −5 [worse] to 5 [better]), current pain medication, sick leave, smoking/moist powder tobacco, and level of education (Supplemental Table 1, http://links.lww.com/PR9/A239). Moist powder tobacco usage was not analysed in this study. In addition, there were 2 questions where the individuals were asked to suggest what they believed was the main reason for being affected by CRPS and one where they were asked if they wanted to convey anything to the researchers conducting the study (Supplemental Table 1, http://links.lww.com/PR9/A239). The free-text questions were not analysed in this study. A reminder was sent out after 2 weeks, and an additional phone contact was made. When appropriate, results were compared with a Swedish reference population (for details, see the result section). The study was approved by the Swedish Ethical Review Authority (No 2021-05570-01, No 2022-00710-02). Research was conducted according to the Declaration of Helsinki and Declaration of the World Medical Association.^60^

### 2.2. Statistics

All categorial variables were presented as n (%), and all scale variables were presented as median (interquartile range; IQR). Any statistical significance between categorical variables was computed by the χ^2^ test. If the cells contained more than 5 counts, the Pearson χ^2^ (exact sig. [2-sided]) was used, and when cells contained less than 5 counts, Fisher exact test was used (exact sig. [2 sided]). In case of a significant *P* value for a categorial variable with 3 groups, each group was compared with another by the χ^2^-test. Statistical significance between categorial and scale variables with 2 individual samples was computed by using a Mann–Whitney *U* test. To analyse the association between the question “rate your disability today compared to when it was at its worst” (dependent variable) and independent variables (type of CRPS, sex, age at diagnosis, level of education, smoking habits, Hospital Anxiety and Depression Scale-Anxiety [HADS-A] score, Hospital Anxiety and Depression Scale-Depression [HADS-D] score, PCS score, and SoC score), a linear regression was made. A reduced model was applied to stepwise eliminate nonsignificant independent variables with a cutoff *P* value of >0.15. A *P* value of 0.05 was accepted as statistically significant. The data were analysed using IBM SPSS Statistics 28.

Concerning a retrospective estimate of the sample size, a minimal clinically important difference for QuickDASH was set at 15.9.^27^ Accordingly, the sample size, with a SD of 28.6, a power (1 − β) at 0.8 and a significance level (α) of 0.05 with the present achieved enrollment ratio of CRPS type 1 and type 2 (n = 71 and n = 28) will be 125 patients, ie, n = 90 and n = 35 in the CRPS groups, respectively (see limitations).

## 3. Results

### 3.1. Demographics, responders, and nonresponders

A total of 99 of 238 patients answered the survey (response rate 42%). Out of the responders, 71 (72%) had CRPS type 1 and 28 (28%) had CRPS type 2 (Fig. 1). When comparing responders to nonresponders, there were no significant differences between sex or type of CRPS. The responders were slightly older than the nonresponders (*P* < 0.001). No significant differences were found between the time since diagnosis and the time since end of follow-up (Table 1).

**Table 1:** Demographics of the total population with complex regional pain syndrome divided into responders and nonresponders of 7 mailed questionnaires.

	Total population (n = 238)	All responders (n = 99)	Nonresponders (n = 139)	*P*
Sex				
Female/male	155 (65)/83 (35)	71 (72)/28 (28)	84 (60)/55 (40)	0.07
Type of CRPS				
Type 1/type 2	158 (66)/80 (34)	71 (72)/28 (28)	87 (63)/52 (37)	0.14
Age at follow-up (y)	56 [46–64]	59 [52–66]	53 [41–62]	**<0.001**
Time since diagnosis (mo)*	60 [34–89]	59 [34–94]	60 [36–86]	0.92
Time since end of follow-up (mo)†	38 [19–65]	36 [20–68]	40 [18–65]	0.94

Data are presented as n (%) or median [IQR]. Bold values indicates *P* < 0.05.

*P* value based on a χ^2^ test for categorical values and Mann–Whitney *U* test for continuous data. In cases with categorical data with cells <5, Fisher exact test was used, and when cells >5, the Pearson χ^2^ test was used.

* Thirteen missing values.

† Eighteen missing values.

CRPS, complex regional pain syndrome; IQR, interquartile range.

### 3.2. Type of Complex Regional Pain Syndrome, improvement of disability, sick leave, medication, and smoking

Individuals with both types of CRPS answered that their disability was reduced over time, CRPS type 1 more than CRPS type 2 (*P* = 0.006; Table 2). No significant differences were found between CRPS type 1 and CRPS type 2 concerning sex, age at follow-up, age at diagnosis, time since diagnosis, time since the end of follow-up, if they currently took any medication for pain, amount of sick leave, smoking habits, and level of education. Twenty-three percent of responders were still on sick leave (only counting those between 18–65 years, n = 71). For those who had been on sick leave for a limited time, the median time was 6.5 [6.0–15.0] months (no difference between type 1 and type 2; Table 2). Paracetamol was the most common pain medication in both groups (43% of total responders) followed by nonsteroidal anti-inflammatory drugs (12%) and opioids (10% whereof 3% heavy opioids, ie, methadone; Table 2). There was no significant difference in smoking habits between the participants in this study and the Swedish general population (current smoker: 1691 [10%], previous smoker 3416 [20%], and never smoked 11 805 [70%], *P* = 0.23; not shown).^26^ There was a difference between this study population and the Swedish general population regarding the level of education (elementary school: 1,485,133 [22%], upper secondary school 3,036,426 [45%], postsecondary school 2,185,926 [33%], *P* = 0.0002; as patients with CRPS had significantly higher education with postsecondary school than normal population; not shown).^56^

**Table 2:** Demographics and complementary questions of responders of 7 questionnaires with complex regional pain syndrome divided into type 1 and type 2.

	All responders (n = 99)	Type 1 (n = 71)	Type 2 (n = 28)	*P*
Sex				
Female/male	71 (72)/28 (28)	54 (76)/17 (24)	17 (61)/11 (39)	0.13
Age at diagnosis (y)*	53 [45–61]	54 [44–63]	52 [47 to 58]	0.44
Age at follow-up (y)	59 [52–66]	59 [47–69]	58 [55 to 63]	0.67
Time since diagnosis (mo)†	59 [34–94]	61 [34–87]	49 [34 to 95]	0.91
Time since last follow-up (mo)‡	36 [20–68]	38 [20–64]	34 [19 to 82]	0.99
Disability today compared with when it was at its worst (worse [−5] − better [5])§	2.0 [0.0–4.0]	3.0 [0.5–4.0]	1.0 [−4.0 to 3.0]	**0.006**
Pain medication today‖				
Yes/no	51 (52)/47 (48)	37 (52)/34 (48)	14 (52)/13 (48)	0.98
If answering yes: what type of pain medication?¶				
Paracetamol	42 (43)	32 (45)	10 (37)	
NSAID	12 (12)	10 (14)	2 (7)	
Opioids	10 (10)	6 (8)	4 (15)	
Heavy opioids (ie, methadone)	3 (3)	1 (1)	2 (7)	
Amitriptyline	6 (6)	5 (7)	1 (4)	
Gabapentin	5 (5)	3 (4)	2 (7)	
Pregabalin	2 (2)	1 (1)	1 (4)	
Antianxiety/antidepressants	6 (6)	4 (6)	2 (7)	
Muscle relaxer	3 (3)	3 (4)	0 (0)	
Dorsal column stimulator	1 (1)	0 (0)	1 (4)	
Undefined pain medication	1 (1)	1 (1)	0 (0)	
Time of sick leave (age 18–65 years, n = 71)#				0.46
No sick leave (0 months)	9 (13)	7 (15)	2 (9)	
Sick leave (>0 months)	44 (64)	31 (66)	13 (59)	
No. of months	6.5 [6.0–15.0]	7.0 [6.0–15.0]	6.0 [3.8–13.3]	0.56
Not applicable (still on sick leave)	16 (23)	9 (19)	7 (32)	
Smoking**				0.90
Current smoker	5 (5)	4 (6)	1 (4)	
Previous smoker	23 (23)	17 (24)	6 (22)	
Never smoked	70 (71)	50 (70)	20 (74)	
Level of education††				0.60
Elementary school	14 (14)	9 (13)	5 (19)	
Upper secondary school	33 (34)	23 (32)	10 (37)	
Postsecondary education	51 (52)	39 (55)	12 (44)	

Data are presented as n (%) or median [IQR]. Bold values indicates *P* < 0.05.

*P* value based on a χ^2^ test for categorical values and Mann–Whitney *U* test for continuous data. In cases with categorical data with cells <5, Fisher exact test was used, and when cells >5, the Pearson χ^2^ test was used.

* Five missing values.

† Six missing values.

‡ Nine missing values.

§ Three missing values.

║ One missing value.

¶ One missing value.

# Three missing values.

** One missing value.

†† One missing value.

IQR, interquartile range; NSAID, nonsteroidal anti-inflammatory drug.

### 3.3. Survey

#### 3.3.1. Disabilities of the Arm, Shoulder and Hand—Quick version and Specific Hand Surgery Questionnaire-8 questions

**Figure 2.**
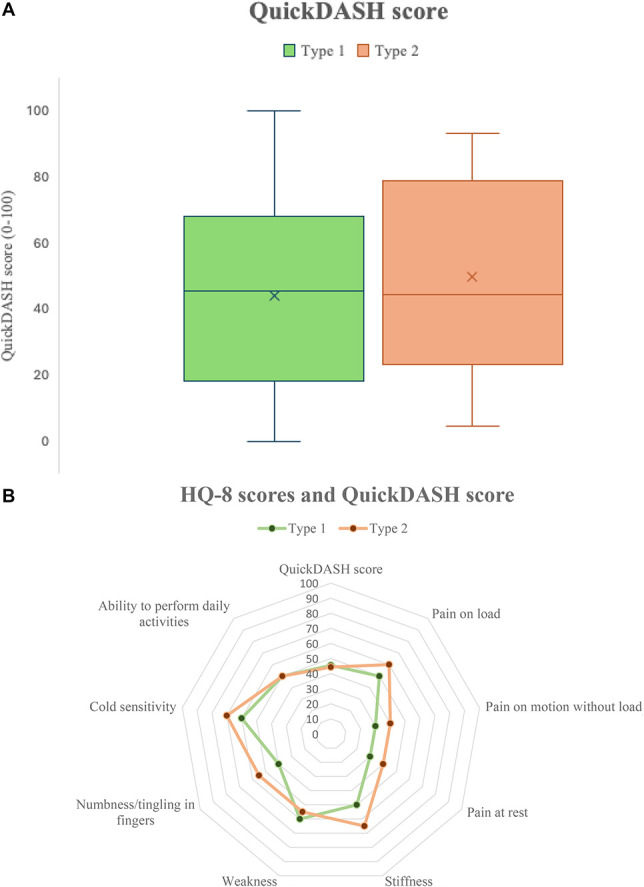
(A and B) Disabilities of the Arm, Shoulder and Hand—Quick (QuickDASH) version and Specific Hand Surgery Questionnaire-8 questions (HQ-8) scores in individuals with CRPS type 1 and type 2. (A) QuickDASH score from individuals with CRPS type 1 and CRPS type 2. Individual scores are presented in Table 3. The X indicates the median value. There was no significant difference in QuickDASH score between individuals with CRPS type 1 compared with individuals with CRPS type 2 (*P* = 0.35). (B) Median HQ-8 questionnaire and QuickDASH scores comparing CRPS type 1 and type 2. Each item is rated on an eleven-grade scale from 0 to 100. Individual values are presented in Table 3. CRPS, complex regional pain syndrome; HQ-8, Specific Hand Surgery Questionnaire-8 questions; QuickDASH, Disabilities of the Arm, Shoulder and Hand—Quick version.

The majority of responders had a disability in their dominant hand/limb, regardless of the type of CRPS. Of all responders, 60 individuals (60%) described limitations when performing work or daily activities (ie, “moderately limited” to “unable”) and 49 individuals (49%) described difficulty sleeping because of their disabled arm, shoulder, or hand (ie, “moderately limited” to “so much that I can't sleep at all”). The average QuickDASH score for responders was 45 (20–70) with no significant difference between CRPS type 1 and type 2 (*P* = 0.35). In the HQ-8 survey, the most prominent symptoms for the total group of individuals were cold sensitivity, pain during loaded exercise, and weakness with no reported difference between patients with CRPS type 1 and type 2 regarding difficulty to sleep, pain, stiffness, weakness, numbness/tingling, cold sensitivity, and ability to perform daily activities (Figs. 2A, B and Table 3).

**Table 3:** Answers from Disabilities of the Arm, Shoulder and Hand—Quick and Specific Hand Surgery Questionnaire- 8 questions questionnaires from patients with complex regional pain syndrome divided into type 1 and type 2.

	All responders (n = 99)	Type 1 (n = 71)	Type 2 (n = 28)	*P*
Disability in the dominant hand*				0.38
Yes	59 (63)	42 (61)	17 (71)	
No	34 (37)	27 (39)	7 (29)	
QuickDASH score (0–100)	45 [20–70]	45 [18–68]	44 [23–79]	0.35
Limited in work or other daily activities (QD)				0.98
Not limited at all	22 (22)	16 (23)	6 (21)	
Slightly limited	17 (17)	12 (17)	5 (18)	
Moderately limited	25 (25)	17 (24)	8 (29)	
Very limited	13 (13)	10 (14)	3 (11)	
Unable	22 (22)	16 (23)	6 (21)	
Difficulties to sleep (QD)†				0.19
No difficulty	32 (33)	27 (39)	5 (18)	
Mild difficulty	17 (17)	10 (14)	7 (25)	
Moderate difficulty	15 (15)	12 (17)	3 (11)	
Severe difficulty	17 (17)	10 (14)	7 (25)	
So much that I can't sleep at all	17 (17)	11 (16)	6 (21)	
Pain (HQ-8)				
At rest	30 [0–60]	30 [0–50]	40 [13–70]	0.07
On motion without load‡	30 [10–60]	30 [0–60]	40 [10–70]	0.13
During loaded exercise	60 [20–80]	50 [20–80]	60 [23–80]	0.48
Stiffness (HQ-8)	50 [20–80]	50 [20–80]	65 [20–88]	0.28
Weakness (HQ-8)	60 [30–80]	60 [30–80]	55 [30–95]	0.87
Numbness/tingling (HQ-8)§	50 [10–70]	40 [0–70]	55 [30–80]	0.05
Cold sensitivity (HQ-8)‖	70 [10–90]	60 [10–80]	70 [20–100]	0.15
Ability to perform daily activities (HQ-8)	50 [10–70]	50 [10–70]	50 [10–78]	0.48

Data are presented as n (%) or median [IQR]. Bold values indicates *P* < 0.05.

*P* value based on a χ^2^ test for categorical values, and Mann–Whitney *U* test for continuous data. In cases with categorical data with cells <5, Fisher exact test was used, and when cells >5, the Pearson χ^2^ test was used.

* Six missing values.

† One missing value.

‡ One missing value.

§ One missing value.

‖ Two missing values.

HQ-8, Specific Hand Surgery Questionnaire-8 questions; IQR, interquartile range; QuickDASH, Disabilities of Arm, Shoulder and Hand- Quick version.

#### 3.3.2. EuroQol 5 Dimensions 3 Levels

Thirty-three of 99 individuals (34%) reported some problems with mobility or being confined to bed. The majority did not have any trouble with self-care (73/99 [74%]), but most individuals had some problems or were unable to perform any usual activities (56/99; 56%). Only 9 individuals (9%) with CRPS reported no pain or discomfort. Of the individuals with CRPS, 49 individuals (50%) described moderate or extreme anxiety or depression. There was no significant difference in any of the dimensions and no difference in overall health between CRPS type 1 and type 2. The individuals with CRPS consistently rated their problems significantly higher than the reference population (Swedish general population aged 20–88 years, *P* < 0.0001) in all 5 dimensions (Table 4).

**Table 4:** Answers from EuroQol 5 Dimensions 3 Levels questionnaire from patients with complex regional pain syndrome divided into patients with type 1 and type 2.

	All responders (n = 99)	Reference population	*P*	Type 1 (n = 71)	Type 2 (n = 28)	*P*
Mobility*			**<0.0001**			0.19
I have no problem in walking about	63 (66)	2727 (89)		47 (69)	16 (57)	
I have some problems in walking about	32 (33)	334 (11)		21 (31)	11 (39)	
I am confined to bed	1 (1)	8 (0)		0 (0)	1 (4)	
Self-care†			**<0.0001**			0.57
I have no problems with self-care	73 (74)	3010 (98)		54 (77)	19 (68)	
I have some problems washing or dressing myself	23 (23)	43 (1)		15 (21)	8 (29)	
I am unable to wash or dress myself	2 (2)	16 (1)		1 (1)	1 (4)	
Usual activities			**<0.0001**			0.21
I have no problems with performing my daily activities	43 (43)	2825 (92)		28 (39)	15 (54)	
I have some problems with performing my usual activities	49 (49)	192 (6)		39 (55)	10 (36)	
I am unable to perform my usual activities	7 (7)	52 (2)		4 (6)	3 (11)	
Pain/discomfort‡			**<0.0001**			0.41
I have no pain or discomfort	9 (9)	1709 (56)		8 (11)	1 (4)	
I have moderate pain or discomfort	66 (67)	1265 (41)		47 (67)	19 (68)	
I have extreme pain or discomfort	23 (23)	95 (3)		15 (21)	8 (29)	
Anxiety/depression§			**<0.0001**			0.21
I am not anxious or depressed	49 (50)	2176 (71)		32 (46)	17 (61)	
I am moderately anxious or depressed	38 (39)	850 (28)		31 (44)	7 (25)	
I am extremely anxious or depressed	11 (11)	43 (1)		7 (10)	4 (14)	
Your health today‖	70 [50–80]¶	Mean 80 (SD 0.8)	NA	71 [50–85]	60 [38–79]	0.09

Data are presented as n (%) or median [IQR]. Bold values indicates *P* < 0.05.

*P* value based on a χ^2^ test for categorical values, and Mann–Whitney *U* test for continuous data. In cases with categorical data with cells <5, Fisher exact test was used, and when cells >5, the Pearson χ^2^ test was used.

Reference population is a study on the Swedish general population aged 20–88 years.^15^

* Three missing values.

† One missing value.

‡ One missing value.

§ One missing value.

‖ Four missing values.

¶ Mean 64.9 (SD 24.7).

IQR, interquartile range; SD, standard deviation.

#### 3.3.3. Life Satisfaction Questionnarie-11

Of all 99 responders, the majority were satisfied in 3 of 11 categories: self-care, family life, and relationships. Around half were satisfied with contact with friends (51%) and life as a whole (49%). Most individuals, regardless of CRPS type, were unsatisfied with their vocation, leisure, sexual life, physical life, and psychological health. Individuals with CRPS type 2 were less satisfied with their physical health compared with patients with CRPS type 1 (*P* = 0.015); otherwise, there was no difference (Table 5).

**Table 5:** Answers from the Life Satisfaction-11 questionnaire from patients with complex regional pain syndrome divided into patients with type 1 and type 2.

	All responders (n = 99)		Type 1 (n = 71)		Type 2 (n = 28)		*P*
	Satisfied	Unsatisfied	Satisfied	Unsatisfied	Satisfied	Unsatisfied	
Life as a whole*	48 (49)	49 (51)	37 (54)	32 (46)	11 (39)	17 (61)	0.20
Vocation†	39 (41)	55 (59)	28 (41)	40 (59)	11 (42)	15 (58)	0.92
Economy‡	45 (47)	51 (53)	31 (46)	37 (54)	14 (50)	14 (50)	0.69
Leisure§	42 (44)	54 (56)	30 (44)	38 (56)	12 (43)	16 (57)	0.91
Contact with friends‖	49 (51)	48 (49)	36 (52)	33 (48)	13 (46)	15 (54)	0.61
Sexual life¶	21 (22)	74 (78)	15 (22)	52 (78)	6 (21)	22 (79)	0.92
Self-care#	61 (63)	36 (37)	44 (64)	25 (36)	17 (61)	11 (39)	0.78
Family life**	69 (73)	25 (27)	49 (74)	17 (26)	20 (71)	8 (29)	0.78
Relationships††	55 (68)	26 (32)	38 (68)	18 (32)	17 (68)	8 (32)	0.99
Physical health‡‡	23 (24)	74 (76)	21 (30)	48 (70)	2 (7)	26 (93)	**0.015**
Psychological health§§	46 (47)	51 (53)	34 (49)	35 (51)	12 (43)	16 (57)	0.57

Data are presented as n (%) with satisfied defined as a score of ≥5 out of 6 ie, a score between 1 and 4 points was defined as unsatisfied. *P* value based on a χ^2^ test for categorical values. In cases with categorical data with cells <5, Fisher exact test was used, and when cells >5, the Pearson χ^2^ test was used. Bold values indicates *P* < 0.05.

* Two missing values.

† Five missing values.

‡ Three missing values.

§ Three missing values.

║ Two missing values.

¶ Four missing values.

# Two missing values.

** Five missing values.

†† Eighteen missing values.

‡‡ Two missing values.

§§ Two missing values.

#### 3.3.4. Hospital Anxiety and Depression Scale

When comparing the 2 types of CRPS for anxiety and depression based on HADS, no significant differences were observed between individuals with CRPS type 1 and type 2 in either category (Table 6). Of the individuals with CRPS type 1, 22 of 71 individuals (31%), and type 2, 10 of 28 individuals (36%), had a prominent score for anxiety (ie, a score of 9–21 points). Of the individuals with CRPS type 1, 17 of 71 (24%), and type 2, 7 of 28 (25%), had a prominent score for depression (ie, a score of 9–21 points). Thus, a significantly higher number of individuals with CRPS had an apparent score for anxiety and depression compared with the reference population (*P* < 0.001; Table 6).

**Table 6:** Answers from the Hospital Anxiety and Depression Scale questionnaire from patients with complex regional pain syndrome divided into patients with type 1 and type 2.

	All responders (n = 99)	Reference population	*P*	Type 1 (n = 71)	Type 2 (n = 28)	*P*
HADS-A score*	6.0 [2.8–10.0]	2 [1–5]		6.0 [2.0–10.0]	5.0 [3.0–10.8]	0.98
HADS-D score†	4.0 [1.0–8.3]	3 [1–5]		4.0 [1.0–8.3]	4.0 [2.0–11.0]	0.53
HADS-A score >8‡	33% (32/99)	10% (697/6659)	**<0.001**	31% (22/71)	36% (10/28)	0.68
HADS-D Score >8§	24% (24/99)	10% (641/6659)	**<0.001**	24% (17/71)	25% (7/28)	0.94

Data are presented as median [IQR] or % (share/total). Bold values indicates *P* < 0.05.

*P* value based on a χ^2^ test for categorical values, and Mann–Whitney *U* test for continuous data. In cases with categorical data with cells <5, Fisher exact test was used, and when cells >5, the Pearson χ^2^ test was used.

The reference population is a general Swedish population, age group 65 to 80 years old.^21^

* One missing value.

† One missing value.

‡ One missing value.

§ One missing value. The values can range from 0 to 12 points in each category. A score over 8 is deemed a significant level of anxiety or depression.

HADS-A, Hospital Anxiety and Depression Scale-Anxiety; HADS-D, Hospital Anxiety and Depression Scale-Depression; IQR, interquartile range.

#### 3.3.5. Pain Catastrophizing and Sense of Coherence-29

Out of all 99 responders, 23 of 97 individuals (24%) had high pain catastrophizing (score >30) and 18 of 89 patients (20%) had a low SoC (score 70–119), with no significant differences in pain catastrophizing and SoC between individuals with CRPS type 1 and type 2. In total, 18 of 69 individuals (26%) with CRPS type 1 had high pain catastrophizing (total score >30) and 5 of 28 individuals (18%) with type 2 (*P* = 0.39). The median SoC score for the total group was 146 (119–163) (not shown). For individuals with CRPS type 1, 22% (14/64) had a low SoC compared with 4/25 (16%) individuals with type 2 (*P* = 0.54). Ten values were missing or deemed nonvalid (>190 or <70 points; Table 7).

**Table 7:** Answers from the Pain Catastrophizing Scale questionnaire and Sense of Coherence-29 questionnaire from patients with complex regional pain syndrome divided into patients with type 1 and type 2.

	All responders (n = 99)	Type 1 (n = 71)	Type 2 (n = 28)	*P*
PCS scores*				
PCS score (0–30 points)/PCS score (31–52 points)	74 (76)/23 (24)	51 (74)/18 (26)	23 (82)/5 (18)	0.39
SoC-29 scores†				
Normal or high SoC (120–190 points)/Low SoC (70–119 points)	71 (80)/18 (20)	50 (78)/14 (22)	21 (84)/4 (16)	0.54

Data are presented as n (%).

*P* value based on a χ^2^ test for categorical values. In cases with categorical data with cells <5, Fisher exact test was used, and when cells >5, the Pearson χ^2^ test was used. Values in the SoC questionnaire range from 29 to 203. A score over 190 or below 70 was defined as nonvalid.

* Two missing values.

† Ten missing values.

PCS, Pain Catastrophizing Scale; SoC-29, Sense of Coherence-29.

### 3.4. Demographics, symptoms, and outcome based on Pain Catastrophizing and Sense of Coherence

There were no differences between individuals with low compared with normal/high SoC or between high and low pain catastrophizing regarding age and sex. Individuals with high pain catastrophizing and individuals with a low SoC rated that their disability today was worse (*P* = 0.002, *P* < 0.001), they had a higher QuickDASH score (*P* < 0.001, *P* < 0.001), were more likely to be unsatisfied with their life as a whole (*P* = 0.003, *P* < 0.001), with their physical health (*P* = 0.012, *P* = 0.008), and with their psychological health (*P* = 0.006, *P* < 0.001) compared with those with low pain catastrophizing and those with a high SoC, respectively (Table 8).

**Table 8:** Demographics, disability, QuickDASH score and results from LiSat-11 questionnaire from patients with complex regional pain syndrome divided into patients with low/high pain catastrophizing and low/normal-high sense of coherence.

	Low PCS score (0–30) (n = 74)	High PCS score (31–52) (n = 23)	*P*	Normal or high SoC (120–190) (n = 71)	Low SoC (70–119) (n = 18)	*P*
Age at diagnosis*	54 [46–63]	48 [43–56]	0.07	55 [45–63]	50 [44–56]	0.31
Sex						
Female/male	53 (72)/21 (28)	17 (74)/6 (26)	0.83	53 (75)/18 (25)	12 (67)/6 (33)	0.50
Rate your disability today compared with when it was at its worst (worse [−5] − better [5])†	3.0 [0.0–4.0]	0.5 [-4.8–2.0]	**0.002**	3.0 [0.0–4.0]	1.0 [(-4.0)-2.0]	**<0.001**
QuickDASH score	36 [18–60]	68 [61–84]	**<0.001**	41 [18–61]	69 [60–84]	**<0.001**
LiSat-11						
Life as a whole Satisfied/unsatisfied‡	42 (58)/31 (42)	5 (22)/18 (78)	**0.003**	45 (63)/26 (37)	0 (0)/18 (100)	**<0.001**
Physical health Satisfied/unsatisfied§	22 (30)/51 (70)	1 (4)/22 (96)	**0.012**	21 (30)/50 (70)	0 (0)/18 (100)	**0.008**
Psychological health Satisfied/unsatisfied‖	40 (55)/33 (45)	5 (22)/18 (78)	**0.006**	43 (61)/28 (39)	0 (0)/18 (100)	**<0.001**

Data are presented as n (%) or median [IQR]. Bold values indicates *P* < 0.05.

*P* value based on a χ^2^ test for categorical values, and Mann–Whitney *U* test for continuous data. In cases with categorical data with cells <5, Fisher exact test was used, and when cells >5, the Pearson χ^2^ test was used.

* Five missing values.

† Three missing values.

‡ One missing value.

§ One missing value.

‖ One missing value.

IQR, interquartile range; LiSat-11, Life Satisfaction Questionnaire-11; PCS, Pain Catastrophizing Scale; QuickDASH, Disabilities of the Arm, Shoulder and Hand—Quick version; SoC, Sense of Coherence.

### 3.5. Linear regression analysis of the question “rate your disability today compared with when it was at its worst”

The selected independent variables, and considered relevant, for analysis using the reduced model (*P* < 0.15) were the type of CRPS, sex, age at diagnosis, level of education, smoking habits, HADS-A score, HADS-D score, SoC score, and PCS score based on the number of responding individuals. When applying the reduced model, there was a significant association between “rate your disability today compared to when it was at its worst” and level of education (Exp B = 1.07, 95% confidence interval (0.32–1.82), *P* = 0.006), and HADS-A score (Exp B = −0.28, confidence interval (−0.39 to −0.17), *P* < 0.001). Thus, a higher level of education was associated with improved disability over time compared with a lower level of education, and a higher HADS-A score (ie, more anxiety) was associated with worsened disability over time compared with a lower HADS-A score (ie, less anxiety).

## 4. Discussion

Individuals with CRPS, the chronic primary pain disorder with defined term nociplastic pain reflected in ICD-11^4,24,25,59^ have problems with pain and sleep as well as limitations during work or daily activities,^37^ but CRPS type 2 shows less improvement than CRPS type 1. Individuals were satisfied with their self-care, family life, and relationships, but approximately 20% to 25% of the individuals had a significant score for depression and anxiety. Pain catastrophizing and a low SoC were associated with worse outcome.

### 4.1. Physical symptoms and disability

There were few differences between individuals with CRPS type 1 and those with type 2, where half of individuals still around 5 years after diagnosis reported a remaining continuous consumption of, mainly light strength, pain medication and one-fourth with persistent sick leave; data were rarely reported in long term in individuals with CRPS.^37^ In other studies, a substantial higher consumption of opioids have been described.^40,53^ Despite this, the present individuals expressed persistent symptoms and disability, like sleep disturbances, and the presence of different pain modalities/discomfort, stiffness, weakness,^5^ numbness/tingling, cold sensitivity, and impaired mobility and physical health as well as decreased ability to perform daily activities and to work based on the present questionnaires with specific questions about physical symptoms and disability. However, without any present observed differences between CRPS type 1 and type 2, our cohort had less moderate-extreme sleeping problems in comparison to previous reports of pain-induced sleep disorders or sleeping problems.^40,53^ Pain modalities, a prominent feature in CRPS, being frequently expressed as moderate to extreme (EQ-5D-3L), showed no difference related to type of CRPS. Problems expressed in HQ-8, which can be related to QuickDASH scores for all individuals and being higher than a similar general age group,^1^ are scores that explain differences in performing daily activities in individuals with CRPS type 1.^51^ Conversely, individuals with CRPS type 1 generally reported more improvement over time concerning their “disability today compared to when it was at its worst” than those with CRPS type 2. Data from Li-Sat-11 questionnaire, where CRPS type 2 individuals were more unsatisfied in their physical health, together with impaired improvement in CRPS type 2 indicate the impact of a concomitant nerve injury for residual problems. Furthermore, several questionnaires may be needed to fully cover the spectrum of symptoms, disabilities, and other aspects in life, thereby revealing any distinction between the 2 types of CRPS. Thus, a majority of individuals with CRPS experience high pain scores, sleep disturbance, opioid use and are not able to perform activities of daily living; problems may differ depending on criteria of CRPS^40^ as well as between countries, indicating the need for studies related to geographical and cultural areas.

### 4.2. Psychological health and other aspects of life

Many present individuals with CRPS had problems in other aspects of their life, such as psychological health, sexual life, and presence of anxiety or depression^2,7,10,11,45,58^ but without any differences between CRPS type 1 and type 2. By contrast, many individuals were satisfied, with some differences from a reference population, with their life as a whole, self-care, family life, relationships, and contact with friends, which is essentially in agreement with surgically treated patients with ulnar nerve entrapment.^29^ However, presence of anxiety or depression was higher compared with a reference population and some published studies^7,21^ but reported to be even higher in other studies depending on used patient-reported outcome measures.^53^ Again, the type of CRPS did not influence the presence of anxiety or depression, indicating no impact of any nerve injury. Depression in individuals with early CRPS is linked with disability and sick leave. Therefore, psychological comorbidities in these individuals are important to identify and address early,^8,43^ an important biopsychosocial aspect.

### 4.3. Pain catastrophizing and sense of coherence

Pain catastrophizing and SoC are novel aspects in evaluating individuals with CRPS.^24^ One-fourth of the responders had high PCS score and one-fifth had a low SoC (compare median score with general Swedish population 151; interquartile range not shown),^39^ which negatively affected outcome. The individuals with high pain catastrophizing and low SoC scores were unsatisfied to a higher extent concerning physical health, including higher QuickDASH scores, but also life as a whole, and the psychological health. High PCS score among individuals with CRPS seems to be linked with depression and kinesiophobia (ie, fear of pain related to movement).^3^ Pain-related fear leads to a worse outcome and may predict functional outcome in CRPS.^7,20^ Evaluating SoC can help to identify individuals more likely to have a worse outcome and ensure extra support early in the disease progression, which is relevant for rehabilitation of patients.^18,35^ The need to address biopsychosocial factors when treating CRPS is supported by our findings and also recently highlighted^24,42^ given that such individuals may be more psychologically dysfunctional than other individuals with chronic pain.^13^ However, this needs to be proven for CRPS by measuring SoC and pain catastrophizing before and after development of the disorder, thus confirming causation. The bidirectional relationship, including cause and effect, between CRPS and mental illness should be addressed in clinical practice.^12^ A biopsychosocial perspective is relevant in intervention strategies in chronic neuropathic pain and in CRPS,^24^ where identification of individuals at risk for CRPS type 1 and type 2 should be identified, particularly among those at risk for poor outcome in long term.^17,47^

### 4.4. Limitations of the study

One limitation is the low response rate, highlighting the statistical power of our sample (see retrospective power calculation in Methods). Patients with CRPS may be “hard to reach” and are a specific population, who at time of the survey are living with the chronic primary pain disorder CRPS,^24^) also having other present problems limiting and justifying the nonresponse. Complex regional pain syndrome is a disease of low prevalence and thus a population of limited access. Working with “hard-to-reach” populations and specific risk groups are relevant because any obtained information is valuable just because of the limited access. We are aware that the measurements may be imprecise, but they provide guidance on a situation of interest. Due to the self-selection bias, there is a limitation for generalizing our findings, and with a risk of that the sample and the target populations may differ. This bias occurs when participants voluntarily choose to participate in our (and in similar) study.

Another problem may be the large number of questionnaires in the survey; thus, a risk of reducing the quality of the responses. We used validated surveys that grouped questions by topic, and several surveys (HADS and SoC-29) switch whether low or high scores results in worse outcome, limiting options to answer every question the same without careful reading. Responding individuals had higher age, a common pattern in register studies.^61^ Interestingly, the assumption of nonresponder bias for mailed surveys was challenged recently among hand surgery patients, indicating that data may still be valid despite a low response rate.^57^

Another limitation is that individuals not speaking Swedish were not able to participate because the surveys were in Swedish. When using multiple choice survey forms, there is a risk of not getting all the information, eg, the sick leave question in the complementary questions was based on a three-level answer and could not determine for what reason the responder was on sick leave or to what level. Furthermore, when asking about pain medication, it is impossible to determine whether the medication is used for pain from CRPS or another cause. In addition, we used an external reference group when available or applicable. There is also a need for standardized outcome questionnaires for CRPS, especially reliable psychometric tools, although the presently used survey provides a “global view” of the diagnosed and treated individuals with CRPS.^31,48^ In addition, there are no biomarkers for CRPS.^11^ A strength is that, despite the response rate, the number of participating individuals is consistent with other follow-up studies on CRPS.^37^ Allowing individuals to answer the surveys anonymously in their own time at home, instead of at their clinic in front of medical staff, limits the response bias. A final limitation is that we were not, from present data in patient folders, able to collect or calculate any CRPS severity score.^34^

In conclusion, individuals with CRPS type 1 and type 2, still at 5 years after diagnosis, suffer from pain, sleeping problems, anxiety, depression, and limitations in daily activities. They are unsatisfied with many aspects of their life, but limited differences between CRPS type 1 and type 2 are found, despite more improvement over time expressed in CRPS type 1. A low SoC or high PCS score among individuals with CRPS type 1 and type 2 are associated with a worse outcome. Using several validated questionnaires in a well-defined population of CRPS is an innovative research perspective.

## Disclosures

The authors have no conflicts of interest to declare.

This work was supported by grants from the Swedish Research Foundation (grant number 2021-01942), Lund University, the Swedish Diabetes Foundation (DIA2020-492), Funds from Skåne University Hospital (2022-974), and by ALF Grants in Region Skåne, Region Östergötland (register number RÖ978765 and RÖ985138), Sweden, and Elly Olsson's Foundation for Medical Research.

The complete and detailed individual data of all subjects cannot be publicly available for ethical and/or legal reasons due to compromising patient privacy based on Swedish law. The National Ethical Committee (https://etikprovningsmyndigheten.se/en/) has imposed these restrictions. Data can be obtained after application and approval of the research project by the National Ethical Committee (https://etikprovningsmyndigheten.se/en/) and by the data safety committees of the regional health care system in Region Skåne, Sweden (KVB-decision; https://vardgivare.skane.se/kompetens-utveckling/forskning-inom-region-skane/utlamnande-av-patientdata-samradkvb/) and in Region Östergötland, Linköping, Sweden.

## Appendix A. Supplemental digital content

Supplemental digital content associated with this article can be found online at http://links.lww.com/PR9/A239.
