# Supplementary material for: Complex Regional Pain Syndrome: a cross-sectional study of physical symptoms, disability, and psychological health in long term
Source: Pain Rep. 2024 Sep 20;9(5):e1180. doi: 10.1097/PR9.0000000000001180 (PMC11419548; doi:10.1097/PR9.0000000000001180)
Supplement: Supplementary file 1 [file painreports-9-e1180-s001.pdf]

Supplemental table 1: Used questionnaires to evaluate patients with CRPS type 1 and CRPS type 2 among 99 responders (questionnaires sent to 238 patients fulfilling Budapest criteria for CRPS; response rate 42%) consisting of CRPS type 1 (72%) and CRPS type 2 (28%) with a median follow up time since diagnosis of 59 [34-94] months

1. Disabilities of the Arm, Shoulder and Hand – Quick version (QuickDASH)

2. Specific Hand Surgery Questionnaire-8 questions (HQ8)

3. EuroQol 5 Dimensions 3 levels (EQ-5D-3L)

4. Life Satisfaction Questionnaire (LiSat-11)

5. Hospital Anxiety and Depression Scale (HADS)

6. Pain Catastrophizing Scale (PCS)

7. Antonovsky's Sense of Coherence-29 (SoC-29)

8. Five complementary questions and two questions with free answers:

a. current disability compared to when it was at its worst [rating -5 (worse) to 5 (better)]
b. current pain medication
c. sick-leave
d. smoking/moist powder tobacco
e. level of education.
f. suggestion on what they believed was the main reason for being affected by CRPS
g. if they wanted to convey anything to the researchers conducting the study. The free-text questions were not analysed in the present study.

For references concerning the questionnaires see Methods.
